# Image quality in ultra-low-dose chest CT versus chest x-rays guiding paediatric cystic fibrosis care

**DOI:** 10.1007/s00330-025-11835-3

**Published:** 2025-07-25

**Authors:** Niamh Moore, Patrick O’Regan, Rena Young, Grainne Curran, Michael Waldron, Alex O’Mahony, Mo’ayyad E. Suleiman, Mary Jane Murphy, Michael Maher, Andrew England, Mark F. McEntee

**Affiliations:** 1 Discipline of Medical Imaging and Radiation Therapy, School of Medicine, University College Cork, Cork, Ireland; 2 Department of Radiology, Cork University Hospital, Cork, Ireland; 3 Sydney School of Health Sciences, University of Sydney, Sydney, NSW Australia; 4 School of Health Sciences, University of Southern Denmark, Odense, Denmark; 5 Faculty of Medicine, University of Sydney, Sydney, NSW Australia

**Keywords:** Children, Lung disease, Diagnostic imaging, follow-up

## Abstract

**Objectives:**

Cystic fibrosis (CF) is a prevalent autosomal recessive disorder, with lung complications being the primary cause of morbidity and mortality. In paediatric patients, structural lung changes begin early, necessitating prompt detection to guide treatment and delay disease progression. This study evaluates ultra-low-dose CT (ULDCT) versus chest x-rays  (CXR) for children with CF (CwCF) lung disease assessment. ULDCT uses AI-enhanced deep-learning iterative reconstruction to achieve radiation doses comparable to a CXR.

**Materials and methods:**

This prospective study recruited radiographers and radiologists to assess the image quality (IQ) of ten paired ULDCT and CXR images of CwCF from a single centre. Statistical analyses, including the Wilcoxon Signed Rank test and visual grading characteristic (VGC) analysis, compared diagnostic confidence and anatomical detail.

**Results:**

Seventy-five participants were enrolled, 25 radiologists and 50 radiographers. The majority (88%) preferred ULDCT over CXR for monitoring CF lung disease due to higher perceived confidence (*p* ≤ 0.001) and better IQ ratings (*p* ≤ 0.05), especially among radiologists (area under the VGC curve and its 95% CI was 0.63 (asymmetric 95% CI: 0.51–0.73; *p* ≤ 0.05). While ULDCT showed no significant differences in anatomical visualisation compared to CXR, the overall IQ for lung pathology assessment was rated superior.

**Conclusion:**

ULDCT offers superior IQ over CXR in CwCF, with similar radiation doses. It also enhances diagnostic confidence, supporting its use as a viable CXR alternative. Standardising CT protocols to optimise IQ and minimise radiation is essential to improve disease monitoring in this vulnerable group.

**Key Points:**

***Question*** *How does chest X-ray (CXR) IQ in children compare to ULDCT at similar radiation doses for assessing CF-related lung disease*?

***Findings*** *ULDCT offers superior IQ over CXR in CwCF. Participants preferred ULDCT due to higher perceived confidence levels and superior IQ*.

***Clinical relevance*** *ULDCT can enhance diagnosis in CwCF while maintaining comparable radiation doses. ULDCT also enhances diagnostic confidence, supporting its use as a viable CXR alternative*.

**Graphical Abstract:**

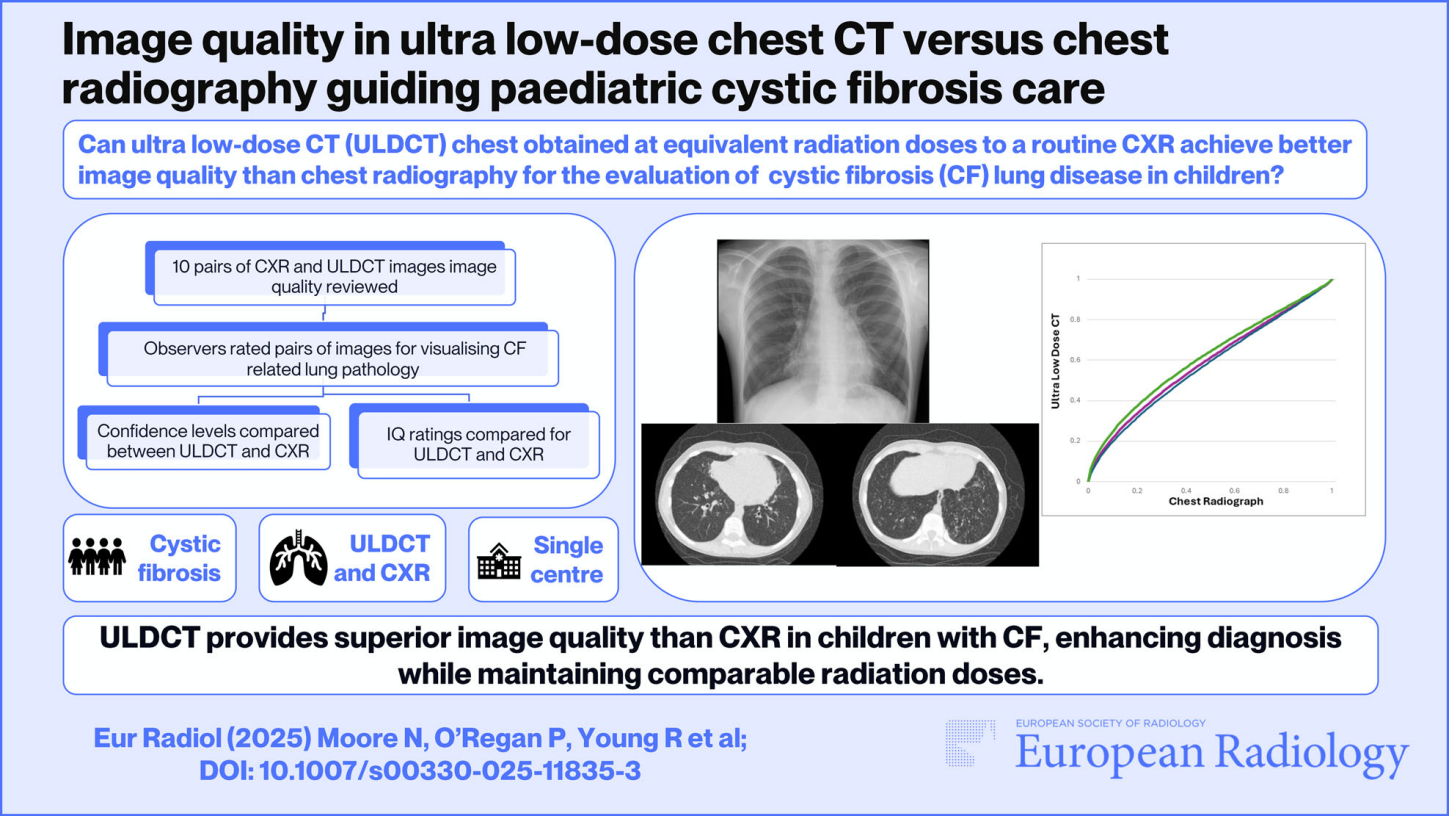

## Introduction

Cystic fibrosis (CF) is the most common life-limiting genetic disorder in the Western World, with pulmonary complications the main cause of morbidity and mortality [1, 2]. Globally, over 162,000 individuals are living with CF [3]. Widespread introduction of newborn screening has led to improved diagnoses in paediatrics [4–7]. Continued efforts to prevent lung disease progression focus on commencing treatments early in life [8] before irreversible lung disease occurs [8–12].

Imaging is crucial in managing CF, monitoring disease progression and assessing treatment efficacy. Chest x-rays (CXR) and computed tomography (CT) play key roles, with CT offering greater sensitivity for detecting early lung changes [1, 13, 14]. MRI has recently also shown great potential for monitoring CF lung disease [1]. However, CT is the gold standard for early CF-associated structural changes [8, 11, 12, 15–17]. Despite its limited sensitivity and specificity, CXR remains ubiquitous [1]and is recommended in clinical guidelines [7, 10]. The heightened susceptibility of children to ionising radiation, along with the associated radiation exposure in CT [18, 19], hinders the routine adoption of CT for CF surveillance [2]. Children are up to ten times more susceptible [18, 20–22] to the effects of radiation compared to adults [23], with estimated effective doses (EDs) for children undergoing chest CT substantially greater than CXR (Table 1) [19].

**Table 1:** Estimated ED of radiation received by children during CXR and chest CT (table adapted/derived from [19])

	ED, mSv				
Examination	0 to < 1 y	1 to < 5 y	5 to < 10 y	10 to < 15 y	15 to < 18 y
Chest radiograph	0.02	0.02	0.01	0.01	0.01
CT thorax	0.57	0.9	1.06	1.69	2.79

In the current paper, “low-dose” denotes radiation levels lower than those used in conventional-dose CT, while “ultra-low-dose CT” (ULDCT) refers to a factorially reduced radiation dose, comparable to, or approximating the exposure of a radiograph for the same anatomical region. The issue of high radiation doses in CT can be potentially addressed with sophisticated algorithms which enhance image quality (IQ), using deep learning iterative reconstruction (DLIR) [24]. Low-dose CT with iterative reconstruction or DLIR is ideal for imaging children with CF (CwCF), given the high inherent contrast in the lungs.

The aim of this study is to assess and compare the IQ of ULDCT of the chest with that of routine CXR, obtained at equivalent radiation doses, for the evaluation of CF lung disease in children.

## Methods

This prospective study obtained ethical approval (Ref number: ECM (uu) 05/12/2023). Observers were asked to compare the IQ of ten ULDCT thorax scans and ten corresponding CXRs from the same CwCF. Informed consent was obtained from all observers.

### Imaging procedure

Between 1st June 2022 and 1st June 2023, a group of 70 CwCF, aged 3–18 years, had both CXR and ULDCT as part of their standard follow-up in a large university teaching hospital with a specialist CF centre. Both imaging procedures had been performed within six weeks of each other. All CwCF had a single PA chest radiograph performed using a Philips Digital Radiography System. CT non-contrast thorax exams were conducted with patients supine, scanning from the apices to the lung bases during arrested inspiration. All CT images were acquired on a Canon Aquilion Prime Sp with advanced intelligent clear IQ engine (AICE) technology (0.5 mm slice thickness, 80 kV, 10 mA, 0.35 s rotation time, standard (0.813) pitch and a 0.10 body CTDI_vol_). All CT images were reconstructed using DLIR. AICE technology used a DLIR algorithm to produce high-contrast CT images at lower radiation doses.

From these 70 CwCF, ten were randomly selected, and their anonymised ULDCT and CXR images were extracted from the Picture Archiving Communications system for IQ analysis. All estimated radiation doses are presented in Table 2.

**Table 2:** Summary of estimated radiation doses for chest CT and CXR for the ten patients’ images, which were reviewed (conversion factors calculated using ICRP 103) [26, 54]

	Chest CT		CXR		
Age (y)	DLP (mGy.cm^2^)	ED (mSv)	Dose area product (Gy.cm^2^)	Tube potential (kVp)	ED(mSv)
5	1.8	0.025	0.058	70	0.009
7	1.9	0.026	0.056	70	0.009
12	2.1	0.029	0.557	125*	0.089
14	1.7	0.024	0.281	125*	0.045
13	1.9	0.026	0.143	70	0.022
7	1.8	0.025	0.078	70	0.012
9	1.8	0.025	0.068	70	0.011
5	1.7	0.024	0.058	70	0.009
13	2.1	0.029	0.521	125*	0.083
14	2.2	0.030	0.422	125*	0.067

* High kV with inclusion of a grid and automatic exposure control

### Observers

For IQ analysis, observers were recruited at the European Congress of Radiology (ECR) in March 2024 and the United Kingdom Imaging and Oncology (UKIO) conference in June 2024. Observers had to be English-speaking and either an experienced CT radiographer, a radiographer in CT training, a radiologist, or a radiologist in CT training. The exclusion criterion was those with no experience in CT.

IQ analysis was performed using an IQ survey (see Supplementary Table 1) that was structured in four sections and hosted on DetectED-X [25]. The survey began with consent and then demographic data collection. In the second section, observers rated CXR quality for visualising CF-related lung pathology and anatomy, using an adaptation of the European paediatric imaging quality criteria [26]. The third section repeated these image quality questions for ULDCT thorax images, using a combined survey adapted from the IQ Criteria for Chest CT in paediatric patients [26] and the European IQ Criteria for the Hi-Res Chest CT. CT images were presented in axial, coronal, and sagittal planes, allowing readers to adjust window settings. Observers rated both sets of images on a scale from 0 to 4, with scores of 0 indicating desired features not seen and 4 indicating higher than needed quality. The final section assessed observers’ confidence in identifying lung pathology using a 5-point Likert scale, asking for their preferred modality for managing CwCF. An additional free-test option was included during UKIO for further comments.

Objective image noise was also measured and quantified to ensure protocol optimisation for acquiring these images. The noise was measured using a validated protocol [18]. A single investigator (NM) with over 20 years of experience in CT conducted the attenuation measurements.

### Data analysis

MS Excel (Microsoft Corporation, Version 16) and IBM SPSS Statistics (Statistical Package for Social Sciences, Version 29) were used for all statistical analyses. The Wilcoxon Signed Rank test was performed, comparing participants’ confidence levels in diagnosing CF-related lung disease using a ULDCT and a CXR and for comparing differences in the paired data of the anatomy visualised on a ULDCT and a CXR. The non-parametric statistical visual grading characteristic (VGC) analyser software (Research Systems, Inc.) [27–31] was used to plot a VGC curve to illustrate IQ ratings for each of the modalities [29]. A one-way ANOVA test was conducted to examine the effect of years of qualification on IQ scores, and a Spearman’s Rho correlation was conducted to assess the relationship between years qualified and IQ scores.

## Results

### Demographics and study setting

**Fig. 1:**
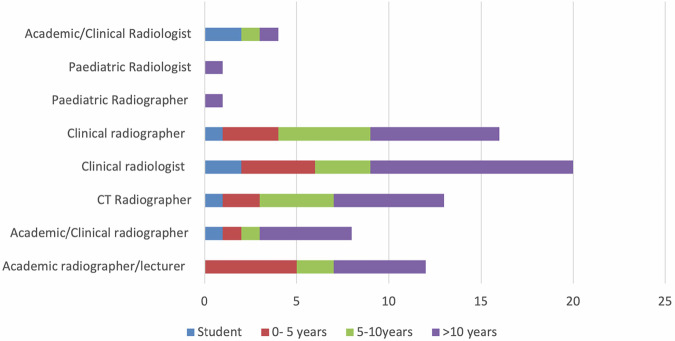
Bar chart summarising the qualification status of observers

A total of 75 observers from 24 countries completed the survey, two-thirds radiographers (*n* = 50) and one-third radiologists (*n* = 25) (Fig. 1). Table 3 summarises the demographics of participants. Ambient lighting levels varied from 5 to 99 lux between three settings (see Supplementary Table 2). The median image noise on the ULDCT was 9.6 ± 4.3 Hounsfield Units (HU) (see Supplementary Table 3).

**Table 3:** Demographics of the observers who completed the image analysis of the ten patients (*n* = 75)

Variable		Total (*n* = 75)
Age (y)	20–30 31–40 41–50 51–60	21 30 19 5
Gender	Male Female	35 40
Role	Academic radiographer/lecturer Academic/clinical radiographer CT radiographer Clinical radiographer Academic radiologist Clinical radiologist Paediatric radiologist Paediatric radiographer	12 8 12 17 4 20 1 1
Qualification experience	Currently a part-time student in postgraduate CT or radiology training Qualified less than 5 y Qualified 5–10 y Qualified for over 10 y	5 17 16 37
Experience of paediatric imaging/reporting	None Some Moderate Extensive	10 38 20 7
Role function	Routinely access images as part of my education role Routinely acquire CT images Routinely acquire and report CT images Routinely report CT images	16 34 8 17

### IQ analysis

**Fig. 2:**
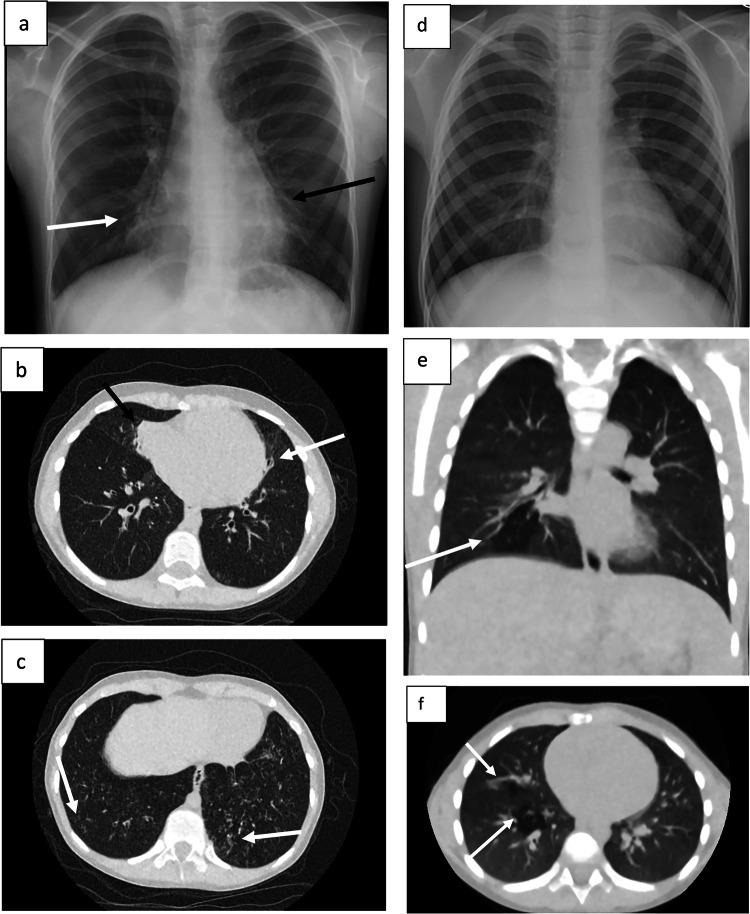
**a**–**c** Plain radiographic and CT features of a 7-year-old with CF. **a** Chest radiograph shows over-inflated lungs with bronchial wall thickening and mild dilatation in the middle lobe (white arrow) and lingula (black arrow). **b** ULDCT shows volume loss in the right middle lobe with subsegmental atelectasis (black arrow) in the medial segment. There is mild bronchiectasis in the lingula (white arrow) and in both lower lobes. **c** ULDCT shows diffuse bronchial wall thickening, mild bronchiectasis and subtle tree-in-bud opacification bi-basally (white arrows). **d**–**f** Plain radiographic and CT features of 5-year-old with CF. **d** Chest radiograph shows no significant abnormalities. **e** ULDCT coronal image showing a focal area of regional hypoattenuation (white arrow) in the right lower lobe, giving a “patchwork appearance”; findings are consistent with mosaic attenuation and suggest air-trapping and small airways disease. **f** ULDCT axial image showing additional areas of mosaic attenuation (white arrow), again suggesting air trapping and diffuse small airways disease. There is also linear opacification in the right middle lobe (white arrow), consistent with plate atelectasis

Eighty-eight per cent of respondents (657/750) reported more confidence in diagnosing CF-related lung disease using ULDCT images than on a CXR (Table 4). Examples of the paired images included in the study can be seen in Fig. 2. The two sets of ULDCT images are samples of high and low IQ. Figure 2a–c scored high IQ with 70/75 observers scoring IQ three (adequate quality) or four (higher than needed), and 55/75 observers gave a low IQ score of zero (desired features not seen), one (unacceptable quality) or two (limited quality) to Fig. 2d–f. The Wilcoxon Signed-Rank test indicated perceived confidence levels were significantly higher (*p* < 0.05) with ULDCT (median = 30) in comparison to CXR (median = 25), *p* < 0.001. Observers were aware that ULDCT images were acquired at comparable radiation doses to CXR, and still, 89% (664/750) of observers preferred ULDCT images to guide patient management (Table 4). When comparing participants’ ability to visualise anatomy, the Wilcoxon signed-rank test indicated no significant difference between ULDCT and CXR (*p* = 0.306).

**Table 4:** Observers responses to the preferred modality for monitoring CwCF

	Image 1	Image 2	Image 3	Image 4	Image 5	Image 6	Image 7	Image 8	Image 9	Image 10
Given that both the CT images and the CXR were acquired at a similar radiation dose, which would you prefer for guiding patient management?										
ULDCT	71	71	64	67	62	70	64	61	66	68
CXR	4	4	11	8	13	5	11	14	9	7
Would you be more confident diagnosing lung disease on the low-dose chest CT or on the chest radiograph?										
ULDCT	71	70	60	69	63	70	61	60	67	66
CXR	4	5	15	6	12	5	14	15	7	9

**Fig. 3:**
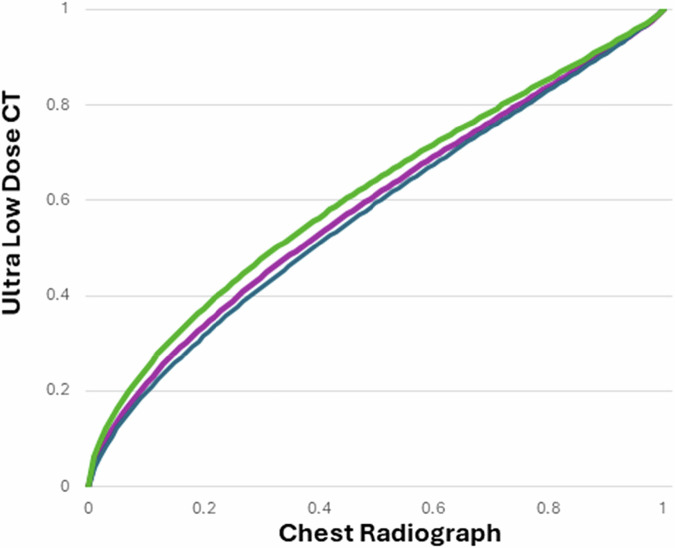
VGC curves rating IQ of ULDCT and CXR to demonstrate lung pathology (bronchiectasis, mucous plugging, pleural effusion, consolidation, ground glass opacities, bullae and atelectasis, and bronchial wall thickening). , Radiologists AUC_VGC_: 0.6262 (Green line). , Combined radiographer and radiologist AUC_VGC_: 0.5880 (Purple line). , Radiographers AUC_VGC_: 0.5710 (Navy line)

VGC analysis was used to compare ULDCT and CXR. VGC curves were obtained by pooling the data from all observers (radiographers and radiologists). The area under the VGC curve (AUC_VGC_) measures the difference in IQ between the two modalities. The combined AUC_VGC_ was 0.59 (asymmetric 95% CI: 0.48–0.68; *p* = 0.08). An AUC of 0.5 indicates modalities are equal. An AUC_VGC_ > 0.5 indicates the modality on the *y*-axis has better IQ. The AUC_VGC_ of 0.59 indicates higher IQ for ULDCT than for the CXR (Fig. 3).

Further VGC analysis was performed on both professions separately. For radiographers, the AUC_VGC_ was 0.57 (asymmetric 95% CI: 0.46–0.68; *p* = 0.20). For radiologists, the AUC_VGC_ was 0.63 (asymmetric 95% CI: 0.51–0.73; *p* = 0.03; Fig. 3).

These AUC_VGC_ values indicate that IQ ratings for demonstrating lung pathology in ULDCT are higher than for CXR. Statistically significant differences (*p* < 0.05) were recorded in the values from the 0.5 value for radiologists, indicating that radiologists scored ULDCT statistically superior for IQ compared to CXR. No statistical differences (*p* > 0.05) were recorded in the AUC_VGC_ values from the 0.5 value when all observers were analysed together or when radiographers were analysed separately.

**Fig. 4:**
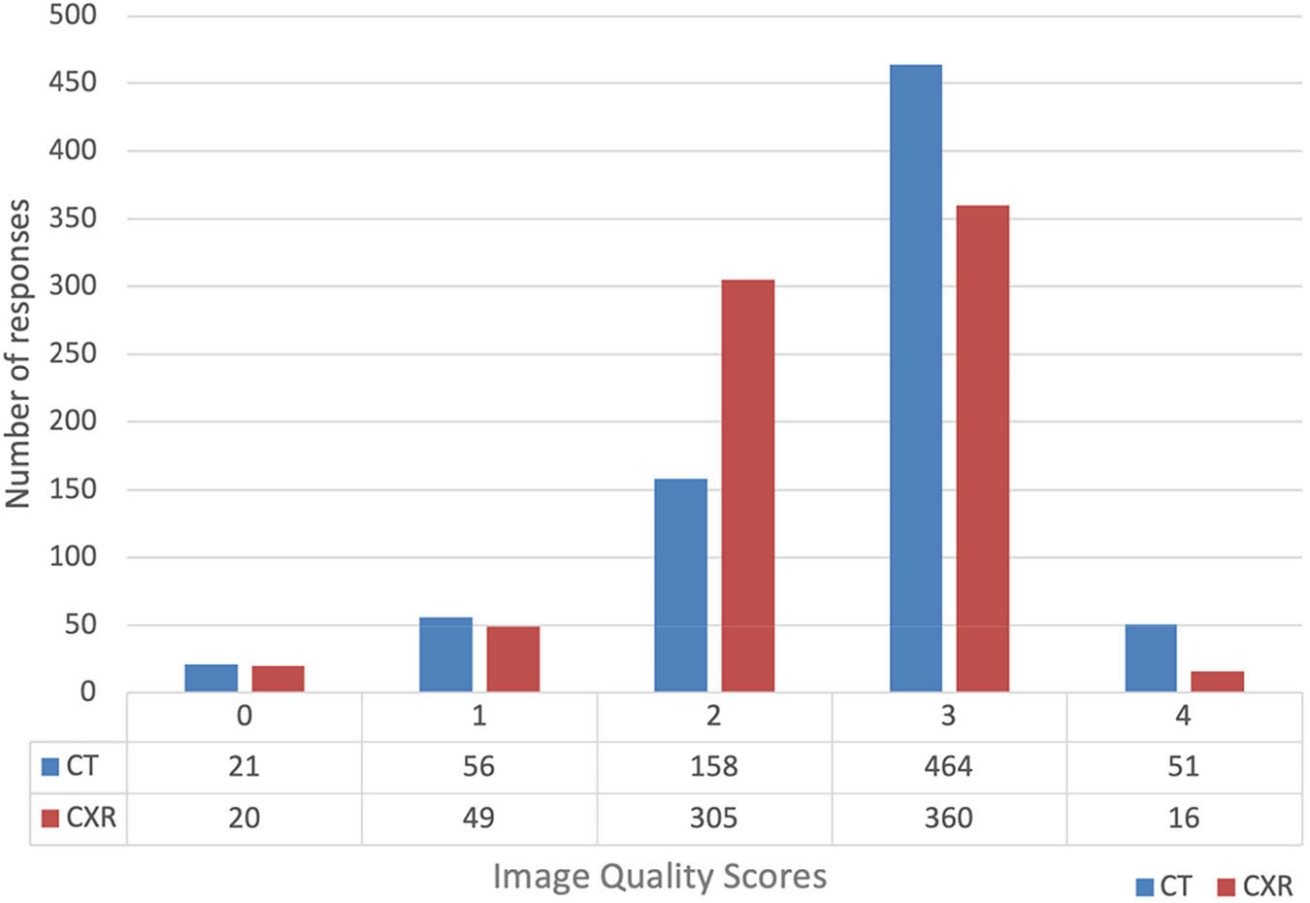
Bar chart demonstrating total IQ scores for ULDCT (blue) and CXR images (red)

The mean IQ score for ULDCT is 2.6 (SD 0.376) while the mean for CXR was 2.4 (SD 0.52). From the overall total for IQ scores, 69% (515/750) rated the ULDCT images as three (adequate quality) or four (higher than needed quality) (Table 5), while 50% (376/750) rated the CXR images as three or four (Fig. 4). For CXR 41% (305/750) rated the CXR as two (limited quality) while 21% (158/750) rated ULDCT as two. Both modalities had similar percentages (ULDCT = 0.02%, CXR = 0.02%) for desired features not seen (zero) and for unacceptable IQ (one) (ULDCT = 0.07%, CXR = 0.06%).

**Table 5:** Summary of the total IQ scores for ULDCT for all 75 observers for each of the images reviewed

IQ rating	0	1	2	3	4
Image 1	5	0	19	49	2
Image 2	2	0	12	53	8
Image 3	1	14	32	27	1
Image 4	2	0	5	59	9
Image 5	4	14	32	24	1
Image 6	0	0	5	60	10
Image 7	2	0	5	58	10
Image 8	2	24	29	19	1
Image 9	0	0	8	64	3
Image 10	3	4	11	51	6

Motion artefact was scored for each ULDCT image, with 72% (539/750) of scores describing the lack of motion artefact as adequate (three) or higher than needed (four). There were no (zero) scores reported missing desired features due to motion artefacts, and only 6% (46 out of 750) reported an unacceptable quality rating (score of one). A recurring theme of the superiority of ULDCT emerged from the nine participants who provided additional comments at the end of the survey (see Supplementary Information Table 4).

### Level of qualification

**Fig. 5:**
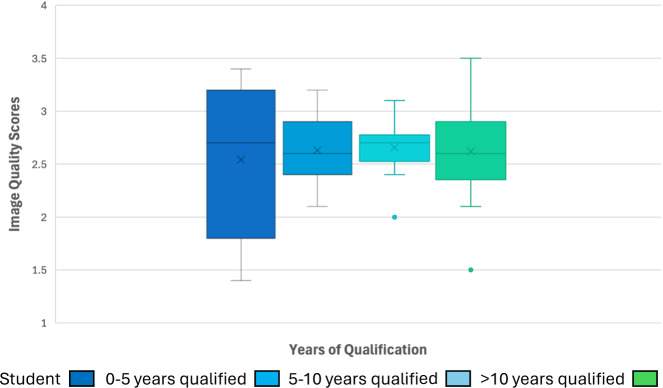
A box and whisker plot representing ULDCT IQ scores (*y*-axis) for each group depending on their years of qualification (*x*-axis). Each box represents the distribution of IQ scores for a particular group of years of qualification. The box itself shows the interquartile range (IQR), which is the range between the first quartile (25th percentile) and the third quartile (75th percentile). The line inside each box represents the median (50th percentile). The X marker inside each box indicates the mean score for that group. The whiskers extending from each box represent the spread of scores beyond the IQR and inside the 5th and 95th percentiles. The dots outside the whiskers represent extremes

Across the years of qualification, similar median values were noted, indicating years of qualification have no effect on IQ scores (Fig. 5). However, the students training in radiology or studying a postgraduate in CT showed the widest range of scores, indicating more inconsistency in IQ evaluation. A Spearman's Rho correlation indicated no significant correlation between years of qualification and IQ scores (0.875). A one-way ANOVA showed no statistical difference between IQ scores for four groups with differing years of qualification F (3, 71) = 0.121, *p*: 0.947. Post-hoc analyses using Tukey's HSD confirmed none of the pairwise comparisons were statistically significant (all *p* > 0.05).

## Discussion

This study demonstrates that ULDCT achieves better IQ results than a CXR when subjective IQ assessment is carried out in CwCF (*p* = 0.03). There is significantly higher perceived confidence using ULDCT (*p* < 0.001). These results highlight the feasibility of replacing traditional CXRs with ULDCT at similar radiation doses, for monitoring lung disease in CwCF.

The development of modulator therapies in 2012 has led to significant improvement in the management of CF disease by treating symptoms and re-establishing proper function of the CFTR protein [10]. Modulator treatments, however, do not reverse structural changes to the airways [5]. Irreversible bronchiectasis precedes abnormalities detectable by traditional methods such as CXRs or spirometry, whereas bronchiectasis, airway wall thickening, mucus plugging, and signs of air trapping, can be detected with CT even in the early stages [1, 14]. CT can identify functional and structural irregularities in both children and adults, even when forced expiratory volume in one second (FEV1) values are normal and there are no other clinical indications of CF disease [8]. The major limitation to using CT is the associated radiation dose; therefore, the exploration of IQ in ULDCT as an alternative to CXR is necessary in this susceptible cohort.

IQ impacts diagnostic accuracy [32], and image noise is the most used physical metric for assessing radiological IQ [33, 34]. The need for optimisation in CT has prompted ongoing efforts to address the challenges of increased noise when reducing dose [35]. In the current study, median image noise on the ULDCT images was 9.6 ± 4.3 HU. Optimal noise should ensure sufficient diagnostic accuracy and radiation dose. High contrast imaging in the CT thorax can tolerate higher noise levels than low contrast imaging, with typical noise levels ranging from 20 HU to 50 HU, so these values are comparable to standard dose CT. EDs ranged from 0.009 mSv to 0.089 mSv for CXR and from 0.024 mSv to 0.030 mSv for ULDCT. DLIR during ULDCT achieved these low noise levels with low radiation doses, reconstructing CT images while preserving noise texture and anatomical structures [36].

Previous research has demonstrated the value of DLIR imaging in the chest, achieving improved IQ and reducing radiation dose [37–39]. This current investigation is the first to evaluate images reconstructed with DLIR specifically in CwCF at radiation dose levels comparable to CXR. With the knowledge that ULDCT and CXR images were acquired at comparable radiation doses, 89% of observers stated a preference to use ULDCT images over a CXR to guide patient management. This was also reflected by observers’ comments, with seven of the additional nine qualitative responses stating ULDCT is superior to CXR. The intricacies of healthcare lie in delivering high-quality services that ensure optimal patient outcomes by selecting the most appropriate patient management [40]. The findings from this study should be integrated into quality improvement initiatives to enhance the care of CwCF.

Over 86% of the respondents had extensive, moderate or some experience in imaging or reporting paediatric CT images and were therefore familiar with IQ in this cohort; only 13% had no experience in paediatric CT. This is important as evaluating clinical IQ is a crucial aspect of optimisation, recognising that diagnostic IQ does not equate to perfect IQ. An IQ score of four in this study represented a quality level exceeding clinical requirements; only 6% rated ULDCT images at this level, suggesting that IQ and consequent radiation doses did not exceed the clinical requirements for this cohort. However, 31% scored the images as limited quality or lower, therefore, there is the potential for IQ improvement. The spread of the overall total IQ scores between CXR and ULDCT followed a similar pattern, however, ULDCT had higher percentages in the highest categories, indicating ULDCT consistently provides better quality for the cases evaluated. CXRs had more responses in the lower quality category (2), which is notable, this may suggest that CT is preferred when high-quality imaging is critical.

The findings of this work illustrated a statistically significant difference in participants’ perceived confidence in making a diagnosis in CwCF, with almost 90% being more confident in using ULDCT thorax than CXR. Appropriate confidence is essential for patient care and management. Existing evidence suggests ULDCT appears superior to CXR in adults with CF [2, 15, 41, 42]. In the current study, only one observer specialised in paediatric image interpretation. It is therefore important to recognise that the observer’s expertise was mainly in adult CT, which may have influenced the perceived confidence and preference levels for use of ULDCT.

The evaluation of IQ is considered subjective and varies between and within groups of radiographers and radiologists [32, 43]. VGC analysis revealed that ULDCT outperformed CXR for IQ assessment for lung pathology. For radiologists, the difference in IQ between the two modalities was significant (*p* = 0.03) while it was not for radiographers, nor for the combined group. Radiologists are the acknowledged experts in image interpretation and may have perceived subtle additional information provided by ULDCT over CXR [44]. Radiologists focus on diagnostic criteria [45] while radiographers are known to evaluate images based on technical criteria [32]. Previous research has also reported variations in clinical judgement between these two groups, with radiologists rating images of higher quality than radiographers [35, 46]. Although radiographers with appropriate postgraduate education can learn to interpret images [47], clinical reporting is an area of advanced practice for diagnostic radiographers. Only one radiographer in the current study reported that they carry out image interpretation. Two-thirds of the observers were radiographers, this majority may account for the lack of statistical significance in the AUC_VGC_ values when all observers were analysed together or when radiographers were analysed separately.

IQ assessment is said to be impacted by years of experience working as a radiographer [48] or a radiologist [49], but similar to Kjelle et al [49], years of qualification were not demonstrated to affect IQ assessment in the results of this study. It is important to recognise that the number of paired images reviewed in this study was small (*n* = 10). However, a power calculation based on a mean of 2.6, SD of 0.376, a minimum detectible effect of 1 and an equivalence margin of 0.1, indicates a power of 98.38%.

When imaging children, the fear component of the X-ray equipment, along with higher respiratory and heart rates, can contribute to motion artefacts in CT. Hospitals are typically seen as intimidating, particularly by children [50]. Despite this, 72% of observers reported that the IQ was adequate or higher than required to show motion artefacts. Such a high IQ is likely attributed to the advanced speed of the latest CT scanners along with the familiarity CwCF have due to frequent hospital visits [44]. This familiarity may partially explain the high IQ results with reduced motion artefacts.

Current ULDCT techniques enable image acquisition with a very low effective radiation dose in comparison to typical EDs for paediatric chest CT 1.1–7.5 mSv [51]. These doses can vary significantly across different centres. Kuo and colleagues also highlighted a significant variation in CT protocols in CwCF in radiation doses and IQ across the 16 centres that they examined [12]. International CF experts in 2019 indicated that a key priority was the ‘Accurate understanding of the risk of radiation with surveillance CT’ [16]. Experimental and epidemiological evidence suggests that cumulative exposure to ionising radiation, even at low doses, heightens the risk of malignancy [19], particularly in younger patients [51, 52]. With improved modulator therapies and earlier diagnosis, CF patients are living longer. However, with longer life expectancy comes certain challenges [5], including increased incidence of certain digestive tract malignancies [52], making cumulative lifetime doses even more relevant. This current research reveals ULDCT has a higher IQ with a significantly lower ED (0.026 mSv, ±0.002), than CXR (0.036 mSv, ±0.03). It is time to change imaging guidance to reflect this.

### Limitations

A CXR was used as a comparator, as conventional dose CT would not be ethically justified in this paediatric population. The exposure parameters in ULDCT remained constant to ensure consistency and to meet ethical obligations controlling the extra radiation dose to this paediatric population. However, it is recommended that imaging protocols be patient-specific to optimise imaging.

As a consequence of recruiting observers at different research hubs, lighting conditions were not equal, with a wide range of illumination levels 5–99 lux, which may have affected the IQ rating, as current recommendations for illumination levels are 25–50 lux [53]. Although 70 patients underwent both the ULDCT and CXR protocols, a key limitation was that, due to time constraints, only ten pairs of images were reviewed by observers. This decision followed a pilot study using 20 image pairs, which required 60–90 min for observers to assess. Based on this, the authors opted to limit the final study to ten pairs to ensure feasibility. Additional work is also recommended to quantify how each observer's preference levels changed regarding IQ for each modality for each image. Finally, all images reviewed were acquired in a single centre study and image review was in a controlled setting rather than a clinical environment, which created a potential for selection bias.

## Conclusion

Standardising CT protocols across centres, focusing on optimising IQ and minimising radiation exposure, is crucial for improving clinical care in this vulnerable population. ULDCT provides superior IQ compared to CXR in CwCF, while maintaining comparable radiation doses. ULDCT also significantly improves diagnostic confidence, highlighting its potential as a viable alternative to CXR. It is time to change imaging guidance to reflect this.

## Supplementary information

ELECTRONIC SUPPLEMENTARY MATERIAL

## Abbreviations

AICE: Advanced Intelligent Clear IQ Engine
AUC_VGC_: Area under the VGC curve
CXR: Chest x-ray
CwCF: Children with cystic fibrosis
CT: Computed tomography
CF: Cystic fibrosis
DLIR: Deep learning iterative reconstruction
ED: Effective dose
ECR: European Congress of Radiology
HU: Hounsfield units
IQ: Image quality
IQR: Interquartile range
ULDCT: Ultra-low dose CT
UKIO: United Kingdom Imaging and Oncology
VGC: Visual grading characteristics
