# Supplementary material for: Image quality in ultra-low-dose chest CT versus chest x-rays guiding paediatric cystic fibrosis care
Source: Eur Radiol. 2025 Jul 25;36(1):586–96. doi: 10.1007/s00330-025-11835-3 (PMC12712111; doi:10.1007/s00330-025-11835-3)
Supplement: Supplementary file 1 — ELECTRONIC SUPPLEMENTARY MATERIAL [file 330_2025_11835_MOESM1_ESM.pdf]

Image quality in Ultra Low-Dose Chest CT versus Chest Radiography guiding paediatric Cystic Fibrosis care

ELECTRONIC SUPPLEMENTARY MATERIAL

Table 1 : Image Quality Questionnaire
DEMOGRAPHICS

1. Please confirm where you are completing this data?
2. What age category are you in?
   a. 20-30 years
   b. 31-40 years
   c. 41-50 years
   d. 51-60 years
   e. 61-70 Years
   f. 71 + Years
3. In which country do you mainly work in?
4. What gender do you identify as ?
   a. Male
   b. Female
   c. Other
5. In terms of years of qualification, which category best describes you?

I am currently a qualified radiographer training to specialise in CT

   a. I am currently a radiologist in training
   b. I have been qualified for less than 5 years
   c. I have been qualified for between 5 and 10 years
   d. I have been qualified for over 10 years
6. In terms of paediatric imaging/reporting which category best describes your experience in imaging or reporting?
   a. No experience
   b. Some experience
   c. Moderate experience
   d. Extensive experience
7. Please select the category that best describes your current or latest employment?
   a. Clinical radiologist
   b. Clinical radiographer
   c. Academic radiologist/lecturer
   d. Academic radiographer/lecturer
   e. Academic/Clinical Radiologist
   f. Academic/Clinical Radiographer
   g. Paediatric Radiologist
   h. Paediatric CT Radiographer
   i. CT Radiographer
   j. Other

8. In relation to CT imaging, which of the following categories best describes your current role?
    a. I routinely acquire CT images
    b. I routinely acquire and report CT images
    c. I routinely report CT images
    d. I access CT images as part of my role in education

Questions for LDCF Case 1,2,3,4,5,6,7,8,9

1. Indicate the most appropriate score for the image quality to demonstrate Cystic Fibrosis lung pathology (bronchiectasis, mucous plugging, pleural effusion, consolidation, ground glass opacities, bullae and atelectasis, bronchial wall thickening) ?
    a. 0s= Desired features not seen
    b. 1= Unacceptable quality
    c. 2=Limited quality
    d. 3=Adequate quality
    e. 4=Higher than needed quality
2. Please indicate how well can you visualise the pulmonary fissures?
    a. 0s = Desired features not seen
    b. 1 = Unacceptable quality
    c. 2 = Limited quality
    d. 3 = Adequate quality
    e. 4 = Higher than needed quality
3. How confident are you be to assess CF lung pathology from the LDCT?
    a. 1= No Confidence
    b. 2= Little Confidence
    c. 3=Confident
    d. 4=Very Confident
    e. 5=Extremely Confident
4. Viewing lung windows Indicate the most appropriate score that describes the image quality to show motion artifacts?
    a. 1 = Unacceptable quality
    b. 2 = Limited quality
    c. 3 = Adequate quality
    d. 4 = Higher than needed quality
5. Please indicate how well can you visualise the entire lung anatomy (including the lung parenchyma, the pulmonary vessels and the bronchi)?
    a. 0s = Desired features not seen
    b. 1 = Unacceptable quality
    c. 2 = Limited quality
    d. 3 = Adequate quality
    e. 4 = Higher than needed quality

Questions for XR Case 1,2,3,4,5,6,7,8,9

1. Please indicate how well can you visualise the trachea and the proximal bronchi ?
    a. 0s= Desired features not seen
    b. 1= Unacceptable quality
    c. 2=Limited quality
    d. 3=Adequate quality
    e. 4=Higher than needed quality
2. Please indicate how well can you visualise the diaphragm and the costo-phrenic angles?
    a. 0s= Desired features not seen
    b. 1= Unacceptable quality
    c. 2=Limited quality
    d. 3=Adequate quality
    e. 4=Higher than needed quality
3. Indicate the most appropriate score for the image quality to demonstrate Cystic Fibrosis lung pathology (bronchiectasis, mucous plugging, pleural effusion, consolidation, ground glass opacities, bullae and atelectasis, bronchial wall thickening) ?
    a. 0s= Desired features not seen
    b. 1= Unacceptable quality
    c. 2=Limited quality
    d. 3=Adequate quality
    e. 4=Higher than needed quality
4. Please indicate how well can you visualise the spine and paraspinal structures and visualise the retrocardiac lung and the mediastinum?
    a. 0s= Desired features not seen
    b. 1= Unacceptable quality
    c. 2=Limited quality
    d. 3=Adequate quality
    e. 4=Higher than needed quality
5. Please indicate the most appropriate score for visualisation of the vascular pattern in central 2/3 of the lungs?
    a. 0s= Desired features not seen
    b. 1= Unacceptable quality
    c. 2=Limited quality
    d. 3=Adequate quality
    e. 4=Higher than needed quality
6. How confident are you be to assess CF lung pathology from the chest radiograph?
    a. 1=No Confidence
    b. 2= Little Confidence
    c. 3= Confident
    d. 4= Very Confident
    e. 5= Extremely confident
7. Would you be more confident diagnosing lung disease on the low dose chest CT or on the chest radiograph?
    a. A low dose Chest CT
    b. Chest radiograph

8. Given that both the CT images and the chest radiograph were acquired at a similar radiation dose which would you prefer for guiding patient management?
    a. A low dose Chest CT
    b. Chest radiograph

Questions for LDCT Case 10

1. Indicate the most appropriate score for the image quality to demonstrate Cystic Fibrosis lung pathology (bronchiectasis, mucous plugging, pleural effusion, consolidation, ground glass opacities, bullae and atelectasis, bronchial wall thickening) ?
    a. 0s= Desired features not seen
    b. 1= Unacceptable quality
    c. 2=Limited quality
    d. 3=Adequate quality
    e. 4=Higher than needed quality
2. Please indicate how well can you visualise the pulmonary fissures?
    a. 0s= Desired features not seen
    b. 1= Unacceptable quality
    c. 2=Limited quality
    d. 3=Adequate quality
    e. 4=Higher than needed quality
3. How confident are you be to assess CF lung pathology from the LDCT?
    a. 1=No Confidence
    b. 2= Little Confidence
    c. 3= Confident
    d. 4= Very Confident
    e. 5= Extremely confident
4. Viewing lung windows Indicate the most appropriate score that describes the image quality to show motion artifacts?
    a. 1 = Unacceptable quality
    b. 2 = Limited quality
    c. 3 = Adequate quality
    d. 4 = Higher than needed quality
5. Please indicate how well can you visualise the entire lung anatomy (including the lung parenchyma, the pulmonary vessels and the bronchi)?
    a. 0s = Desired features not seen
    b. 1 = Unacceptable quality
    c. 2 = Limited quality
    d. 3 = Adequate quality
    e. 4 = Higher than needed quality
6. For each of the cases the chest radiograph and LDCT have been acquired at a similar radiation dose, have you any additional comments you would like to add?

    FREE TEXT RESPONSE

Questions for XR Case 10

1. Please indicate how well can you visualise the trachea and the proximal bronchi ?
    a. 0s= Desired features not seen
    b. 1= Unacceptable quality
    c. 2=Limited quality
    d. 3=Adequate quality
    e. 4=Higher than needed quality
2. Please indicate how well can you visualise the diaphragm and the costo-phrenic angles?
    a. 0s= Desired features not seen
    b. 1= Unacceptable quality
    c. 2=Limited quality
    d. 3=Adequate quality
    e. 4=Higher than needed quality
3. Indicate the most appropriate score for the image quality to demonstrate Cystic Fibrosis lung pathology (bronchiectasis, mucous plugging, pleural effusion, consolidation, ground glass opacities, bullae and atelectasis, bronchial wall thickening) ?
    a. 0s= Desired features not seen
    b. 1= Unacceptable quality
    c. 2=Limited quality
    d. 3=Adequate quality
    e. 4=Higher than needed quality
4. Please indicate how well can you visualise the spine and paraspinal structures and visualise the retrocardiac lung and the mediastinum?
    a. 0s= Desired features not seen
    b. 1= Unacceptable quality
    c. 2=Limited quality
    d. 3=Adequate quality
    e. 4=Higher than needed quality
5. Please indicate the most appropriate score for visualisation of the vascular pattern in central 2/3 of the lungs?
    a. 0s= Desired features not seen
    b. 1= Unacceptable quality
    c. 2=Limited quality
    d. 3=Adequate quality
    e. 4=Higher than needed quality
6. How confident are you be to assess CF lung pathology from the chest radiograph?
    a. 1=No Confidence
    b. 2= Little Confidence
    c. 3= Confident
    d. 4= Very Confident
    e. 5= Extremely confident
7. Would you be more confident diagnosing lung disease on the low dose chest CT or on the chest radiograph?
    a. A low dose Chest CT
    b. Chest radiograph

8. Given that both the CT images and the chest radiograph were acquired at a similar radiation dose which would you prefer for guiding patient management?
    a. A low dose Chest CT
    b. Chest radiograph

Table 2: Summary of study lighting and display conditions. A calibrated DrMeter Digital Lux Meter (LX1330B) measured ambient light levels in lux at 0.3 meters from each monitor. Images were reviewed at UKIO in a research hub and at ECR in two separate settings.

	ECR Research Hub	ECR- Image Perception Lab	UKIO Research Hub
Lighting Conditions	35 Lux	5-7 Lux	99 Lux
Display Monitor	22.5” Liquid Crystal display office monitor (FlexScan EV2360)	22.5” Liquid Crystal display office monitor (FlexScan EV2360)	32 inch 8MP Barco Medical Display monitor (Eonis 8MP)
No. of Participants	34	24	17

Table 3: Attenuation values for each case observed

Case No.	Noise SD 1	Noise SD 2	Noise SD 3	Mean Value	Median Value
1	8.6	7.8	7.9	8.1	7.9
2	10.2	9.7	12.8	10.9	10.2
3	10.6	11.5	9.7	10.6	10.6
4	20.2	3.0	15.5	12.9	15.5
5	8.5	9.0	9.0	8.8	9.0
6	10.6	14.9	11.2	12.3	11.2
7	6.6	10.8	4.5	7.3	6.6
8	8.1	24.1	18.3	16.8	18.3
9	3.9	3.4	8.1	5.1	3.9
10	5.1	8.6	6.8	6.8	6.8
Average	9.2	10.3	10.4	9.7	9.6

Table 4: Summary of additional comments

PARTICIPANT NUMBER	ADDITIONAL COMMENTS

Participant 1: Movement artefact seems to be the biggest change in quality. Lung windows are invaluable
Participant 4: As dose doesn't always equate to risk or consider radiosensitivity, especially not in CT, it may be useful to look at whether the benefit of CT outweighs the possible increased risk to eyes, thyroid, stomach for example. Unsure if this has been proven as such (may be a cultural assumption that no longer counts with improved tech) but as the helical/scatter over-ranging may cross into these structures (or go close to the eyes) in CT whereas XR is more controlled/collimated to the area we are viewing.
Participant 7: ULDCT sensitivity appears superior
Participant 8: For comparable dose, the 3D advantage of CT makes it superior and gives more confidence in diagnosis, even where IQ is not ideal. Efforts would need to be made to ensure patient compliance with breath holds relative to a CXR.
Participant 13: Overall, CT offers better cross-sectional detail by far (but we already know this). The fact that the radiation dose is the similar to a CXR is just brilliant!
Participant 14: ULDCT better for patients
Participant 15: ULDCT is more valuable
Participant 16: As the doses for the acquired images are similar, it is a simple decision that the low dose CT is much more useful and beneficial for the patient's management and I believe this should become the standard practice.
Participant 17: ULDCT superior
